# Qualitative research methods in medical dissertations: an observational methodological study on prevalence and reporting quality of dissertation abstracts in a German university

**DOI:** 10.1186/s12874-020-01186-6

**Published:** 2020-12-10

**Authors:** Charlotte Ullrich, Anna Stürmlinger, Michel Wensing, Katja Krug

**Affiliations:** Department of General Practice and Health Services Research, University of Heidelberg Hospital, INF 130.3, 69120 Heidelberg, Germany

**Keywords:** Medical dissertation, Higher education, Qualitative research, Methodological study, Reporting

## Abstract

**Background:**

Qualitative methods offer a unique contribution to health research. Academic dissertations in the medical field provide an opportunity to explore research practice. Our aim was to assess the use of qualitative methods in dissertations in the medical field.

**Methods:**

By means of a methodological observational study, an analysis of all academic medical dissertations’ abstracts between 1998 and 2018 in a repository databank of a large medical university faculty in Germany was performed. This included MD dissertations (Dr. med. (dent.)) and medical science dissertations (Dr. sc. hum.). All abstracts including “qualitativ*” were screened for studies using qualitative research methods. Data were extracted from abstracts using a category grid considering a) general characteristics (year, language, degree type), b) discipline, c) study design (mixed methods/qualitative only, data conduction, data analysis), d) sample (size and participants) and e) technologies used (data analysis software and recording technology). Thereby reporting quality was assessed.

**Results:**

In total, 103 abstracts of medical dissertations between 1998 and 2018 (1.4% of *N* = 7619) were included, 60 of MD dissertations and 43 of medical sciences dissertations. Half of the abstracts (*n* = 51) referred to dissertations submitted since 2014. Most abstracts related to public health/hygiene (*n* = 27) and general practice (*n* = 26), followed by medical psychology (*n* = 19). About half of the studies (*n* = 47) used qualitative research methods exclusively, the other half (*n* = 56) used mixed methods. For data collection, primarily individual interviews were used (*n* = 80), followed by group interviews (*n* = 33) and direct observation (*n* = 11). Patients (*n* = 36), physicians (*n* = 36) and healthcare professionals (*n* = 17) were the most frequent research participants. Incomplete reporting of participants and data analysis was common (*n* = 67). Nearly half of the abstracts (*n* = 46) lacked information on how data was analysed, most of the remaining (*n* = 43) used some form of content analysis. In summary, 36 abstracts provided all crucial data (participants, sample size,; data collection and analysis method).

**Conclusion:**

A small number of academic dissertations used qualitative research methods. About a third of these reported all key aspects of the methods used in the abstracts. Further research on the quality of choice and reporting of methods for qualitative research in dissertations is recommended.

## Background

Qualitative research methods offer a unique contribution to health research, particular for exploration of the experiences of patients, healthcare professionals and others [1–5]. While (general) epidemiology primarily addresses health and healthcare in populations and clinical research concentrates on medical interventions and health prognosis, qualitative research methods focus on different actors’ perspectives, experiences and behaviours in health-related contexts. Qualitative research entails a broad spectrum of methods of data conduction and data analysis: individual interviews illuminate individual perceptions [6], group interviews deliver insights into shared norms and opinions [7], direct observations facilitate understandings of behaviours in healthcare practice [8–10] and documents can offer insights into discourses and self-representations [11]. For data analysis, methods combining inductive and deductive steps are most suitable for exploratory research questions utilizing existing results, theories and concepts [12]. Given these prospects, little is known on the practice of applying qualitative research methods, especially concerning medicine.

In dissertations, a foundation for future scientific work is laid; therefore, guidance and rigour are of special importance [13]. Dissertations in medical departments provide a good opportunity to explore research practices of students and young academics. In Germany, about 60% of all graduating medical students complete an academic dissertation [14], which they usually finish parallel to medical school within a full-time equivalent of about a year [15–18]. As a by-product, medical doctoral students are increasingly among the authors of published research, holding first-authorship in about 25% [18–20].

In Germany, basic scientific training is a required part of the medical curriculum and recent policies put even more emphasis on the development of scientific competencies [15, 21, 22]. National regulations specify scientific competencies giving explicit recommendations for quantitative methods. Medical students have rarely received training in qualitative methods. However, health care professions and qualitative methods share a perspective directed to practice and interactions. Interviews and observations are already commonly used as clinical and diagnostic tools.

In addition to the doctoral degrees for medical and dental graduates (Doctor medicinae (dentariae), Dr. med. (dent.)), students with other disciplinary backgrounds (e.g. natural scientists, psychologists and social scientists) complete dissertations at medical faculties in Germany (often labelled Doctor scientiarum humanarum, Dr. sc. hum. or Doctor rerum medicarum, Dr. rer. medic.). Although regulations differ slightly, the degrees are usually situated within and regulated by the same institutional culture and context (e.g. faculty, department, supervision and aspired publications).

The aim of this study was to understand the current practice of applying qualitative research methods helping identify gaps in reporting and need for guidance. By means of a methodological study – a subtype of observational studies that evaluates the design, analysis of reporting of other research-related reports [23] – we investigated volume and variety of the use of qualitative research methods in dissertations at a German medical faculty. Hereby we wanted to inform methodological advances to health research and outline implications for medical education in scientific competencies training.

## Methods

### Search strategy

Dissertations in the medical field were retrospectively assessed: In a document analysis, all dissertation abstracts at one medical faculty were reviewed. This faculty was chosen as it is one of the oldest and largest medical faculties in Germany, with a strong research tradition and a high dissertation rate among graduating students. All abstracts from 01/01/1998 to 31/12/2018, which were publicly available in the repository databank of the university, were reviewed. This included MD dissertations (Dr. med. (dent.)) and medical science dissertations (Dr. sc. hum.) written in German or English. All types of studies using qualitative research methods, all types of human participants, all types of interventions and all types of measures were eligible. We focused on abstracts, because full text dissertations are not publicly available and are helpful to get an overview of a number of method-related issues. Although serving as a proxy, abstracts should provide a sufficient summary of the dissertation, including crucial information on study design, independently from the full text. In the databank, relevant documents had to be labelled a) “abstract of a medical dissertation” (referring to both degree types). To further identify dissertations using qualitative methods b) the search term “qualitativ*” was used as an inclusion criterium.

### Selection and data extraction

All identified abstracts were pre-screened independently by two researchers (AS, LS) and then reviewed by the main research team (KK, AS, CU) excluding abstracts using “qualitativ*” only in respect to non-methods-related issues (e.g. quality of life). Data on a) general characteristics (year, language, degree type), b) discipline, c) study design (mixed methods/qualitative only, data conduction, data analysis), d) sample (size and participants) and e) technologies used (data analysis software and recording technology) (see App. 2) was then extracted independently by two team members (AS, LS) and crossed-checked (KK, CU). Data extraction was initially guided by two widely used reporting guidelines for qualitative health research articles [24, 25] and adapted to reflect the abstract format: Abstracts provided comparable information on the set-up of study design and sample. Reporting of results was not assessed due to heterogeneity and briefness. Data extraction forms were piloted and adjusted to inductive findings. Disagreements were discussed, assessed and solved by consensus by the main research team (KK, AS, CU). Extracted data were analysed and reported as absolute and relative frequencies. As all abstracts were available, no further data was obtained from authors.

## Results

### Search results

Out of a total of 7619 dissertation abstracts, 296 dissertations were initially identified. Of these, 173 abstracts were excluded from the study as “qualitativ*” in these abstracts did not refer to the research method. Additionally, 20 abstracts (12 medicine, 8 medical science) were not further included in the analysis due to an ambiguous and inconclusive use of the label “qualitative methods” and/or restricted comparability with the otherwise pre-dominant interview-based study designs: a) a qualitative research design was stated, but no further information on the approach was given (*n* = 7), b) no explicit distinction was made between qualitative research design and a clinical diagnostic approach (*n* = 4), c) the qualitative approach comprised of additional free text answers in written questionnaires only (*n* = 6), and d) only document analysis or observation was used (*n* = 3). In total, 103 abstracts (1.4% of 7619) were included in the analysis.

### Low but increasing use

**Fig. 1:**
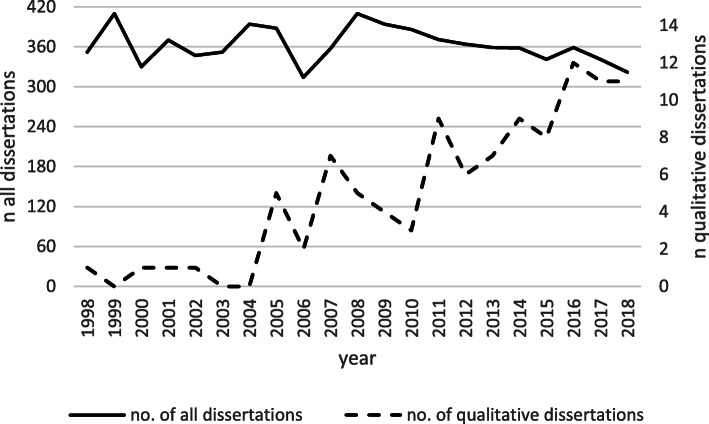
Number of all dissertations and dissertations using qualitative methods per year between 1998 and 2018

Since 1998, the number of dissertations applying qualitative methods has continually increased while the total number of dissertations remained stable (between *n* = 314 in 2006 and *n* = 410 in 1999 and 2008, M (1998–2018) = 362.8, SD = 26.1) (Fig. 1). Before 2005 there was yearly not more than one dissertation that used qualitative methods. Since then, the number has steadily raised to more than 10 dissertations per year, equivalent to an increase from 0.28% in 1998 to 3.42% in 2018 of all listed dissertation abstracts per year.

### General characteristics

Abstracts nearly equally referred to dissertations leading to an MD degree (Dr. med. *n* = 57, Dr. med. Dent. *n* = 3) and medical science degree (Dr. sc. hum. *n* = 43), respectively. The included dissertation abstracts were based in 12 different *sub-specialties*, most in general practice (*n* = 26), in public health and hygiene (*n* = 27) and medical psychology (*n* = 19); the Dr. med. (dent.) abstracts having a higher share in general practice (*n* = 21) and the Dr. sc. hum. abstracts in public health/hygiene (*n* = 16) (s. Table 1).

**Table 1:** Usage of qualitative research design in dissertations at a medical faculty

	Dr. med. (dent.) (*n* = 60)	Dr. sc. hum. (*n* = 43)	Total (*n* = 103)
**A. Department/ Discipline (n %)**			
Public Health/ Hygiene	11 (18.3)	16 (37.2)	27 (26.2)
General Practice	21 (35.0)	5 (11.6)	26 (25.2)
Medical Psychology	7 (11.7)	12 (27.9)	19 (18.4)
Internal Medicine/ Psychosomatics	11 (18.3)	3 (7.0)	14 (13.6)
Health Services Research	3 (5.0)	1 (2.3)	4 (3.9)
Psychiatry	0 (−)	3 (7.0)	3 (2.9)
Paediatrics	3 (5.0)	0 (−)	3 (2.9)
Medical Biometry und Informatics	0 (−)	2 (4.7)	2 (1.9)
Others (Anatomy, Gynaecology, History, Neurology, Social Medicine)	4 (6.7)	1 (2.3)	5 (4.9)
**B. Study Design**			
Mixed Methods Approach	24 (40.0)	32 (74.4)	56 (54.4)
Qualitative Approaches only	36 (60.0)	11 (25.6)	47 (45.6)
**C. Method Data Conduction (multiple indications possible)**			
Document analysis	3 (5.0)	2 (4.7)	5 (4.9)
Interviews (individual)	43 (71.7)	37 (86.0)	80 (77.7)
Group Interviews	21 (35.0)	12 (27.9)	33 (32.0)
Observation	5 (8.3)	6 (14.0)	11 (10.7)
Questionnaire	2 (3.3)	1 (2.3)	3 (2.9)
Not reported	1 (1.7)	4 (9.3)	5 (4.9)
**D. Participants (multiple indications possible)**			
Health Care Professionals	11 (18.3)	6 (14.0)	17 (16.5)
Patients	19 (31.7)	17 (39.5)	36 (35.0)
Physicians	27 (45.0)	9 (20.9)	36 (35.0)
Relatives	4 (6.7)	3 (7.0)	7 (6.8)
Students	10 (17)	1 (2.3)	11 (10.7)
Other	6 (10)	10 (23.3)	16 (15.5)
Not reported	1 (1.7)	6 (14.0)	7 (6.8)
**E. Sample Size**			
*E1 Interviews: Participants*			
2–10	6 (10.0)	4 (9.3)	10 (9.7)
11–20	5 (8.3)	7 (16.3)	12 (11.7)
21–30	9 (15.0)	4 (9.3)	13 (12.6)
31–40	5 (8.3)	3 (7.0)	8 (7.8)
41–50	0 (−)	3 (7.0)	3 (2.9)
51–60	4 (6.7)	2 (4.7)	6 (5.8)
> 60	2 (3.3)	1 (2.3)	3 (2.9)
Not reported	12 (20.0)	13 (30.2)	25 (24.3)
Not used or unclear	17 (28.3)	6 (14.0)	23 (22.3)
*E2 Groups: Number of interview groups*			
1–5	7 (11.7)	1 (2.3)	8 (7.8)
6–10	4 (6.7)	4 (9.3)	8 (7.8)
> 10	2 (3.3)	3 (7.0)	5 (4.9)
Not reported	8 (13.3)	4 (9.3)	12 (11.7)
Not used or unclear	39 (65.0)	31 (72.1)	70 (68.0)
*E3 Groups: Total participants in group interviews*			
2–20	4 (19.0)	1 (8.3)	5 (15.2)
21–40	4 (19.0)	1 (8.3)	5 (15.2)
41–60	3 (14.3)	1 (8.3)	4 (12.1)
61–76	2 (9.5)	0 (−)	2 (6.1)
Not reported	8 (38.1)	9 (75.0)	17 (51.5)
**F. Recording technology used**			
Audio	21 (35.0)	10 (23.3)	31 (30.1)
Video	5 (8.3)	1 (2.3)	6 (5.8)
Not reported	34 (56.7)	32 (74.4)	66 (64.1)
**G. Method of Data Analysis**			
Content Analysis (without P. Mayring)	16 (26.7)	10 (23.3)	26 (25.2)
Content Analysis following P. Mayring	14 (23.3)	2 (4.7)	16 (15.5)
Grounded Theory	1 (1.7)	4 (9.3)	5 (4.9)
Other	6 (1.0)	4 (9.3)	10 (9.7)
Not reported	23 (38.3)	23 (53.5)	46 (44.7)
**H. Qualitative Data Analysis software used**			
ATLAS.ti	12 (20.0)	2 (4.7)	14 (13.6)
MAXQDA	3 (5.0)	0 (−)	3 (2.9)
NVivo	1 (1.7)	2 (4.7)	3 (2.9)
Other	2	1 (2.3)	3 (2.9)
Not reported	42 (70.0)	38 (88.4)	80 (77.7)

Most abstracts followed at least roughly the common structure of background, methods, results and conclusion. The length of the abstracts varied between less than one and more than three pages, with most abstracts being one to two pages long; 77 abstracts were written in German and 26 in English.

### Study design

About half of the studies used qualitative research methods exclusively (*n* = 47; 60% of Dr. med. (dent.) abstracts, 26% of Dr. sc. hum. abstracts), the other half mixed methods (*n* = 56; 40% of Dr. med. (dent.) abstracts, 74% of Dr. sc. hum. abstracts; Table 1). Individual interviews were the most common form of data collection (*n* = 80), followed by group interviews (*n* = 33) and observation (*n* = 11). In total, 23 abstracts indicated the use of a combination of different qualitative methods of data conduction, all of these included individual interviews. For documentation/recording, when reported (*n* = 37), audio recording was used in most cases (*n* = 3).

Little difference regarding method of data conduction were found between pure qualitative and mixed-methods designs. Mixed methods studies rather included physicians (*n* = 21) and used predominantly general content analysis (*n* = 14), when reported; whereas qualitative studies rather included patients (*n* = 28) and used predominantly both content analysis (*n* = 14) and content analysis following Mayring (*n* = 12). Overall incomplete reporting was more common in mixed-method studies (*n* = 41) than qualitative studies (*n* = 26, 55.3%) (see App. 2).

### Sample

*Sample size* varied widely: Overall, 67 abstracts provided a sample size. Of those, a median number of 29 people (min-max: 2–136) participated in individual and group interviews. Only in Dr. sc. hum. dissertations using mixed methods, lower median sample sizes were reported for the qualitative part (Md = 22, min-max: 6–110; *n* = 17) compared to dissertations using qualitative methods only (medical science (*n* = 7): Md = 31, min-max: 16–50; MD (*n* = 29): Md = 29, min-max: 7–136) and Dr. med. (dent.) dissertations with mixed methods (Md = 30, min-max: 2–62; *n* = 14). In individual interviews, when sample size was reported (*n* = 55, 69% of 80), it distributed roughly equally in the ranges of 1–10, 11–20, 21–30, 31–50 and above 50 (Md = 25; min-max: 2–110). For the 33 dissertations using group interviews, the number of groups is given in 20 abstracts, the number of participants in 15 abstracts. Between 1 and 24 group interviews were conducted with a median total of 24 participants (min-max: 2–65) (see Table 1).

Patients (*n* = 36) and physicians (*n* = 36) were the overall most frequent *research participants*, followed by other health care professionals (*n* = 17), students (*n* = 11) and relatives of patients (*n* = 7). Other participants (*n* = 16) included: representatives of self-help organizations and other experts, educators such as teachers and policy makers. In 33% (*n* = 31) of the abstracts, more than one participant group was included, 6.8% (*n* = 7) did not specify research participants. While MD dissertations predominantly included physicians (*n* = 27) and patients (*n* = 19), Dr. sc. hum. dissertations included mostly patients (*n* = 17) and other participants (*n* = 10).

### Data analysis

For data analysis, if reported (*n* = 57), content analyses were the most common used method (*n* = 42), including the highly deductive approach formulated by Mayring [26] (*n* = 16), mostly used in MD dissertations (*n* = 14). Among other reported methods (*n* = 15), grounded theory (*n* = 5) was the most common approach; rarely mentioned methods include framework analysis and non-specific analysis combining inductive and deductive approaches. Forty-six abstracts did not provide information on the analysis method used (38.3% of MD abstracts, 53% of medical science abstracts). If reported (*n* = 24), ATLAS.ti (*n* = 14), MAXQDA (*n* = 3) and NVivo (*n* = 3) were mentioned most frequently as qualitative data analysis programs.

### Reporting

In summary, 36 abstracts provided all crucial data (participants: sample size, characteristics, i.e. healthcare professional/patient; data collection and analysis method). Thus, 58% (*n* = 35) of MD dissertation abstracts and 74% (*n* = 32) of Dr. sc. hum. dissertation abstracts had at least one missing information.

## Discussion

The results show a low but increasing use of qualitative research methods in medical dissertations. Abstracts nearly equally referred to dissertations leading to an MD degree and medical science doctorate respectively; half of which were submitted since 2011. Qualitative methods were used in several departments, most frequently in those for general practice, public health and medical psychology mirroring an already known affinity between the objective of certain medical disciplines and perspective qualitative methods [27, 28].

About half of the studies used qualitative research methods exclusively, the other half mixed methods: While some differences were found, due to short format and sparse information within the abstract a strict differentiation between qualitative approaches alone and combined quantitative and qualitative designs was not made. Little difference according to degree type was observed. This points to a strong shared dissertation culture, that balances and conceals differences in academic training between medical students and graduates from other, quite diverse, disciplines (e.g. from humanities, natural and social sciences) pursuing a doctorate at a medical faculty.

### Limited variety in methods used

The results show a *strong preference for certain methods* in data conduction, research participants and data analysis: Individual and group interviews were predominant as well as content analysis, especially Mayring’s deductive approach. All in all, a limited use of the broad spectrum of qualitative research methods can be observed. Interviews are important to gain insights on actors’ perspective [6]; however, they have limited information value when it comes to actual processes and practice of health care. To investigates those, additional direct observation would be suitable [8, 9]. In group interviews shared norms and opions can be observed, they are not suitable to capture individual perspectices. Group interviews go along with higher time and efforts regarding scheduling, interview guidance and data analysis [7]. Within the dissertations, documents are rarely used as data within the dissertations, but could be useful readily available documents.

Included research participants were mostly patients and physicians. This might be due to the research questions posed or the availability of participants. However, to reflect the complexity of health care a higher diversity of research questions, expanding participants (e.g. other health care professionals and caregivers) and based on a thorough knowledge of available methods, including qualitative approaches, might be needed.

As for methods of analysis, the results show a predominant use of a form of content analysis, with a strong affinity to quantitative analysis often limited to description forgoing in-depth analysis. As qualitative methods belong to the interpretative paradigm, most qualitative methodologies emphasize inductive analyses (e.g. Grounded Theory) and/or a combination of induction and deduction [12, 29]. By using primarily descriptive content analysis the full potential of qualitative research and depth of the data to gain new a insights are thus neglected. Since knowledge about and application of qualitative methods are not part of the medical curriculum, doctoral students lack training in using qualitative methods and grasping the possibilities these methods convey for in-depth original knowledge.

### Incomplete reporting

One fundamental principle of good research practice is accurate reporting. For empirical research, reporting on research design and methods is crucial to ensure comparability and reflect reach of research results. Within medicine and other health sciences, while debated [30], reporting guidelines are increasingly used to guarantee a basic standard. While qualitative research designs differ from clinical and quantitative designs regarding theoretical and methodological background, study aims and research process, rigorous reporting is a shared standard: this includes reporting on data conduction, sampling, participants and data analysis (e.g. COREQ [24]).

In our study, *incomplete reporting* regarding research design and methods was common. Especially, information on methods of data analysis was missing in about half of the abstracts reflecting the limited awareness of the plethora of qualitative analysis methods. Additionally, a third of the abstracts did not provide information on sample size. Although the importance of a “sufficient” sample size is controversially discussed, identifying the sources and putting their contributions into perspective is a paramount characteristic of qualitative research [31–33]. All in all, incomplete reporting was common (*n* = 67). Additionally, out of 123 initially identified abstracts, 20 had to be excluded from the analysis as comparability was not given mainly due to the inconclusive use of the term qualitative methods.

### Limitation

Several issues should be considered when interpreting the findings from this study. As a case study at one large faculty, which has a strong research orientation, the generalizability of the findings is uncertain. It seems unlikely that the quality of reporting is better in other medical faculties in Germany, but the prevalence of using qualitative methods might be higher. Character and role of the abstracts might not be as apparent as in journal papers, as they serve as a summary of the dissertation and are listed within the online repository databank only. The relation of reporting quality of those abstracts and the full text dissertation or even publication is unknown. Presentation of results was not assessed as information in abstracts were brief and heterogenous. Additionally, insights are limited by the structure of the repository databank itself, i.e. sub-disciplines are combined that sometimes cover distinct research fields or did evolve as separate specialties. All in all, however, the results mirror the critique on the lack of scientific training in medical education [17, 22, 34, 35] and the want of sufficient reporting in medicine and health science, irrespective of study design [36].

While recent policies put a strong emphasis on strengthening scientific competences in medical education in Germany [15, 21, 22], especially MD dissertations are only in some degree comparable to dissertation thesis of other disciplines and medical dissertations internationally: In Germany, about 60% of all graduating medical students complete an academic dissertation [14], which they usually finish parallel to medical school within a full-time equivalent of about a year [15–18]. Graduate programs that exclusively dedicate 1 year for pursuing a dissertation are still discussed as innovative [35]. Additionally, the expertise of supervisors was not assessed. In a recent opinion paper, Malterud et al. [37] called for supervisors and dissertation committees holding corresponding methodological skills and experience as well as an academic consensus regarding scientific rigour to ensure high quality theses using qualitative methods. Missing standards in supervision and reporting might have led to the observed results in our study.

## Conclusion

Qualitative research methods offer a unique scientific benefit to health care, including medicine. Our results show that within dissertation research, the number of dissertations applying qualitative methods has continually increased mirroring an overall trend in health research. To improve reach and results, a broader spectrum of qualitative methods should be considered when selecting research designs, including e.g. (direct) observation, document and analysis strategies, that combine inductive and deductive approaches. Same holds true for including a more diverse body of research participants. More broadly, reporting and academic practice should be improved.

Reporting guidelines can not only help to improve the quality of reporting but also be used as a tool to supervising graduate students steps commonly associated with qualitative research methods. So far, however, reporting guidelines mainly target full and/or published papers. Still, some reporting guidelines for abstracts are already available [38–41], that could be adapted for dissertation research in health science.

In academic practice, skilled supervision alongside transparent and method-appropriate criteria are the precondition for confident and courageous dissertation research that increases understanding and challenges existing knowledge of health care research. Educational programs strengthening research and reporting skills – within and beyond qualitative methods – should be implemented into medical education more profoundly, at the latest in doctoral training.

## Data Availability

The data of the repository databank is semi-public and can be accessed from the corresponding author upon reasonable request. A data extraction table is available in App. 2.Competing interests and funding: We have no conflicts of interest to disclose and no funding to report.

## Abbreviations

COREQ: Consolidated criteria for reporting qualitative research
Dr. med.: Doctor medicinae
Dr. med. dent.: Doctor medicinae dentariae
Dr. sc. hum.: Doctor scientiarum humanarum
MD: Medical Doctor

## References

[1] Lewin S, Glenton C (2018). Are we entering a new era for qualitative research? Using qualitative evidence to support guidance and guideline development by the World Health Organization. Int J Equity Health.

[2] Sale JEM, Thielke S (2018). Qualitative research is a fundamental scientific process. J Clin Epidemiol.

[3] Salmon P, Young B (2018). Qualitative methods can test and challenge what we think we know about clinical communication – if they are not too constrained by methodological ‘brands’. Patient Educ Couns.

[4] Tugwell P (2018). Systematic Review Qualitative Methods Series reflect the increasing maturity in qualitative methods. J Clin Epidemiol.

[5] Sibeoni J (2020). A specific method for qualitative medical research: the IPSE (inductive process to analyze the structure of lived experience) approach. BMC Med Res Methodol.

[6] Dicicco-Bloom B, Crabtree BF (2006). The qualitative research interview. Med Educ.

[7] Wilkinson S (1998). Focus groups in health research: exploring the meanings of health and illness. J Health Psychol.

[8] Vindrola-Padros C, Vindrola-Padros B (2018). Quick and dirty? A systematic review of the use of rapid ethnographies in healthcare organisation and delivery. BMJ Qual Saf.

[9] Catchpole K (2017). Framework for direct observation of performance and safety in healthcare. BMJ Qual Saf.

[10] Ullrich C, Oetting-Roß C, Niederberger M, Finne E (2020). Qualitative Beobachtung als Methoden in den Gesundheitswissenschaften. Forschungsmethoden in der Gesundheitsförderung und Prävention.

[11] Bowen Glenn A (2009). Document analysis as a qualitative research method. Qual Res J.

[12] Emerson, R.M., R.I. Fretz, and L.L. Shaw, Writing ethnographic fieldnotes. [2nd publ.] ed. Chicago guides to writing, editing and publishing. 2011. Chicago [u.a.]: Univ. of Chicago Pr.

[13] Dai S (2020). Evaluation of the reporting quality of observational studies in master of public health dissertations in China. BMC Med Res Methodol.

[14] Bundesamt S (2019). Anzahl universitärer Abschlüsse in der human- und Zahnmedizin in Deutschland nach Prüfungsart im Jahr 2018.

[15] Fakultätentag M (2017). Positionspapier Vermittlung von Wissenschaftskompetenz im Medizinstudium.

[16] Diez C, Arkenau C, Meyer-Wentrup F (2002). Bearbeitung und Betreuungsqualität medizinischer Dissertationen an der Medizinischen Fakultät Würzburg aus der Sicht von Promovenden des 5. Und 6. Studienjahres. Gesundheitswesen.

[17] Education, W.F.o.M (2015). Basic Medical Eduation: WFME Global Standards for Quality Improvement: Basic Medical Education.

[18] Pabst R, Park DH, Paulmann V (2012). Die promotion in der Medizin ist besser als ihr Ruf. Dtsch Med Wochenschr.

[19] Cursiefen C, Altunbas A (1998). Contribution of medical student research to the Medline-indexed publications of a German medical faculty. Med Educ.

[20] Ziemann E, Oestmann J-W (2012). Publikationen von Doktoranden 1998–2008. Dtsch Arztebl Int.

[21] BMBF (2017). Masterplan Medizinstudim 2020.

[22] Empfehlungen zur Weiterentwicklung des Medizinstudiums in Deutschland auf Grundlage einer Bestandsaufnahme der humanmedizinischen Modellstudiengänge (Drs. 4017–14), Juli 2014, G.C.o.S.a. Humanities., Editor. 2014.

[23] Mbuagbaw L (2020). A tutorial on methodological studies: the what, when, how and why. BMC Med Res Methodol.

[24] Tong A, Sainsbury P, Craig J (2007). Consolidated criteria for reporting qualitative research (COREQ): a 32-item checklist for interviews and focus groups. Int J Qual Health Care.

[25] O’Brien BC (2014). Standards for reporting qualitative research: a synthesis of recommendations. Acad Med.

[26] Mayring, P., Qualitative Content Analysis. 2000. 2000. 1(2).

[27] Sielk M, Brockmann S, Wilm S (2004). Qualitative Forschung - Hineindeuten in oder Abbilden von Wirklichkeit?. Z Allg Med.

[28] Jaye C (2002). Doing qualitative research in general practice: methodological utility and engagement. Fam Pract.

[29] Gale NK (2013). Using the framework method for the analysis of qualitative data in multi-disciplinary health research. BMC Med Res Methodol.

[30] Barbour RS (2001). Checklists for improving rigour in qualitative research: a case of the tail wagging the dog?. BMJ.

[31] Vasileiou K (2018). Characterising and justifying sample size sufficiency in interview-based studies: systematic analysis of qualitative health research over a 15-year period. BMC Med Res Methodol.

[32] Malterud K, Siersma VD, Guassora AD (2016). Sample size in qualitative interview studies:guided by information power. Qual Health Res.

[33] Hennink MM, Kaiser BN, Weber MB (2019). What influences saturation? Estimating sample sizes in focus group research. Qual Health Res.

[34] Ratte A, Drees S, Schmidt-Ott T (2018). The importance of scientific competencies in German medical curricula - the student perspective. BMC Med Educ.

[35] Thimme R (2019). Strukturierte Karrierewege in der Universitätsmedizin. Dtsch Med Wochenschr.

[36] Zenz M, Zenz J, Grieger M (2018). Erwähnung des Ethikvotums in deutschen medizinischen Dissertationen und Publikationen. Bundesgesundheitsbl Gesundheitsforsch Gesundheitsschutz.

[37] Malterud K, Hamberg K, Reventlow S (2017). Qualitative methods in PhD theses from general practice in Scandinavia. Scand J Prim Health Care.

[38] Cohen JF (2017). STARD for Abstracts: essential items for reporting diagnostic accuracy studies in journal or conference abstracts. BMJ.

[39] Hopewell S (2008). CONSORT for reporting randomized controlled trials in journal and conference abstracts: explanation and elaboration. PLoS Med.

[40] Collins GS (2015). Transparent reporting of a multivariable prediction model for individual prognosis or diagnosis (TRIPOD): the TRIPOD statement. Ann Intern Med.

[41] Bougioukas KI (2019). Reporting guidelines on how to write a complete and transparent abstract for overviews of systematic reviews of health care interventions. J Clin Epidemiol.

